# Supplementary material for: The relationship between the living environment and remote working: an analysis using the SHEL model
Source: PeerJ. 2024 May 7;12:e17301. doi: 10.7717/peerj.17301 (PMC11086296; doi:10.7717/peerj.17301)
Supplement: Supplemental Information 3 [file peerj-12-17301-s003.pdf]

在宅勤務の状況および在宅勤務時の生活環境についてのアンケート

下記の注意事項をよく読んでお答えください。

このアンケートは，「在宅勤務者にとって快適かつ負担が少ない在宅勤務環境の人間工学ガイドラインについての研究：在宅勤務状況と作業空間・生活環境についての実態調査」の一環で実施するものです。アンケートの回答時間はおよそ15~20分程度です.
以下に説明する内容をよく理解していただいたうえで，アンケートの回答にご同意いただけるかどうかをご自身の自由意思において決定してください。

1. 研究の目的
この研究の目的は，就労者の在宅勤務状況，作業環境や生活環境を調査することで，在宅勤務の満足度，生産性，ストレス反応，ワークエンゲージメントに関連する居住環境の要因を明らかにし，在宅勤務者の快適さ・負担低減に寄与する人間工学的知見を得ることを目的としています。そのために，このアンケートではあなたの在宅勤務に対する満足度やあなたご自身の仕事の内容や状況、仕事に関して感じていること、あなたの健康状態、在宅勤務の状況や環境などに関してお伺いする設問が含まれます。
業務上の守秘義務により回答できない場合は、アンケートに参加しなくても問題ありません。また、途中で回答を止めても問題ありません。

2. 調査方法
アイブリッジ株式会社の提供するアンケートツールFreesyを利用したオンライン質問紙調査によって在宅勤務およびその居住環境に関する就労者の生活状況を調査いたします。

3. 期待される効果
本研究は在宅勤務時の種々の居住環境要因と在宅勤務者のストレス反応，満足度，生産性，ワークエンゲージメントなどとの関連性を調査するものであり，在宅勤務時の有効・快適な作業環境構築のための人間工学ガイドラインの検討に貢献することが期待されます。

4. 予想される副作用
本研究によって個人の不利益や危険が起こるような副作用は予見されません。

5. 研究から生じる知的財産について
この研究の結果として特許権などが生じる可能性がありますが、その権利は研究遂行 者に属し、あなたには属しません。また、その特許権などをもととした経済的利益が生じる可能性がありますが、あなたはこれについて権利がありません。

6. 同意されない場合でも不利益は受けません
あなたにこの研究の内容を紹介して、あなた自身で参加の可否を決めていただくための説明同意文書です。内容を正しく理解したうえで、あなた自身の自由な意見によって、この試験の参加を決めてください。この調査の内容や意義、その他の条件などについて、より具体的に知りたい場合、不明瞭な点がある場合は、どんな事でも遠慮なくお問合せください。できる限り質問に答えます。なお、この調査の説明文を読んだ後にこの調査への参加を断っても、あなたに不利益を被ることは一切ありません。

7. 同意した後でもいつでも途中でやめることができます
この調査に参加されるかどうかは、あなたの自由意思でお決め下さい。これを読んだ時点でも、調査参加に同意した後でも、たとえ回答中であっても、いつでもこの調査への参加を止めることができます。そのためにあなたが不当な扱いや不利益を受けることは一切ありません。必要があれば、この調査の参加について、自由に第三者の意見を求める事もできます。

8. プライバシーは守られます
今回の調査を通して取得するデータは、学会、研究会、専門雑誌等に発表したりすることがあります。このような場合には絶対にあなたの名前等がわからないように配慮し、知り得た事実は関係者以外に漏れないように約束致します。なお，データを発表する場合は，統計的に処理されたデータとして扱われます。

実施責任者：大阪公立大学生活科学研究科居住環境学分野　講師　土井俊央
問い合わせ先：
06-6605-2888
tdoi@omu.ac.jp

以上の，説明をよく読み，以下の条件の下で本研究の対象者として参加することに同意いただける場合は，同意のチェックボックスにチェックをしたうえで，アンケート回答へお進みください.
・研究の実施、研究成果の発表に際して、アンケート回答者の人権が尊重され、アンケート回答者の個人情報に関して機密が守られる。
・アンケート回答者の個人情報は、説明を受けた研究目的以外に用いられない。
・アンケート回答者の安全性に関して、十分な配慮及び対策と適切な処置が取られる。
・アンケート回答者が説明を受けた研究計画に基づいて実施される。万一、何らかの変更があった場合には、如何なる場合でもアンケート回答者への説明が行なわれ、アンケート回答者の合意を得る。
・アンケート回答者が説明を受けた測定項目以外の測定は、行なわれない。
・アンケート回答者に疑問や質問が生じた場合には、適切な説明がなされる。
・アンケート回答者に不都合が生じた場合あるいは研究に疑義が生じた場合、アンケート回答者の意志で研究の中断及び研究への参加を中止できる。
・アンケート回答者はアンケート回答者個人に関する情報、データなどについて知る権利がある。

☐ 上記内容に同意する 必須

引き続き、アンケートにご協力いただけますか。

はい

いいえ

COPYRIGHT © iBRIDGE Corporation. All Rights Reserved.

締切	**所定の回答数が集まった時点でアンケートは終了します。あらかじめご了承ください。**

注意事項

※回答はお1人様1回迄です。
※必ず［アンケート完了］ボタンを押して回答を完了してください。
※JavaScriptを有効にしてください。

推奨ブラウザ

Microsoft Edge、Mozilla Firefox、Google Chrome、Safari

アンケート内の情報について

アンケート内容については、いかなる情報も複製、販売、出版、公開などにより他者へ漏洩することを禁止いたします。
ご承諾いただける場合のみ回答にお進みください。

アンケートデータの取り扱いについて

アンケートデータの取り扱いについて当アンケートでご回答いただいた内容は、法規制や当社のプライバシーポリシーに従い、個人名やユーザー名を除いた上で集計データ及びテキストデータとしてアンケート依頼主に開示・提供いたします。

アンケートに回答する

COPYRIGHT © iBRIDGE Corporation. All Rights Reserved.

1

ご自身の職務として近いものを選んでください. （ひとつだけ）*回答必須

○ 非管理職
○ 管理職

次へ

COPYRIGHT © iBRIDGE Corporation. All Rights Reserved.

2

在宅勤務に対する満足度をうかがいます．最もあてはまるものを選んでください． ***回答必須**

		1．非常に満足していない	2．あまり満足していない	3．どちらともいえない	4．やや満足している	5．非常に満足している
在宅勤務という働き方に対する総合的な満足度	→	○	○	○	○	○
在宅勤務時の物理的な室内の仕事環境に対する満足度	→	○	○	○	○	○

次へ

COPYRIGHT © iBRIDGE Corporation. All Rights Reserved.

3

ここ1か月のご自身の仕事の状況についてお答えください. ***回答必須**

		1．そう思わない	2．あまりそう思わない	3．どちらともいえない	4．ややそう思う	5．そう思う
ご自身の仕事の生産性は高いと思いますか	→	○	○	○	○	○
自分は期待されている以上の成果を出していると思いますか	→	○	○	○	○	○
在宅勤務を行うことによって仕事の効率が上がったと思いますか	→	○	○	○	○	○

次へ

COPYRIGHT © iBRIDGE Corporation. All Rights Reserved.

4

次の質問文は，仕事に関してどう感じているかを記述したものです．各文をよく読んで，あなたが仕事に関してそのように感じているかを判断してください．そのように感じたことが一度もない場合には，0（ゼロ）を，感じたことがある場合にはその頻度に当てはまる数字を（1から6）をご回答ください． ***回答必須**

		0：全くない	1：ほとんど感じない	2：めったに感じない	3：時々感じる	4：よく感じる	5：とてもよく感じる	6：いつも感じる
仕事をしていると，活力がみなぎるように感じる	→							
仕事に熱心である	→							
私は仕事にのめりこんでいる	→							

次へ

COPYRIGHT © iBRIDGE Corporation. All Rights Reserved.

5

最近1か月間のあなたの状態についてうかがいます．最もあてはまるものをご回答ください． ***回答必須**

		1．ほとんどなかった	2．ときどきあった	3．しばしばあった	4．ほとんどいつもあった
1.活気がわいてくる	→				
2.元気がいっぱいだ	→				
3.生き生きする	→				
4.怒りを感じる	→				

		1．ほとんどなかった	2．ときどきあった	3．しばしばあった	4．ほとんどいつもあった
5.内心腹立たしい	→	○	○	○	○
6.イライラする	→	○	○	○	○
7.ひどく疲れた	→	○	○	○	○
8.へとへとだ	→	○	○	○	○
9.だるい	→	○	○	○	○
10.気がはりつめている	→	○	○	○	○

		1．ほとんどなかった	2．ときどきあった	3．しばしばあった	4．ほとんどいつもあった
11.不安だ	→	○	○	○	○
12.落ち着かない	→	○	○	○	○
13.ゆううつだ	→	○	○	○	○
14.何をするのも面倒だ	→	○	○	○	○
15.物事に集中できない	→	○	○	○	○
16.気分が晴れない	→	○	○	○	○
17.仕事が手につかない	→	○	○	○	○
18.悲しいと感じる	→	○	○	○	○
19.めまいがする	→	○	○	○	○

	1.ほとんどなかった	2.ときどきあった	3.しばしばあった	4.ほとんどいつもあった
20.体のふしぶしが痛む →	○	○	○	○

	1.ほとんどなかった	2.ときどきあった	3.しばしばあった	4.ほとんどいつもあった
21.頭が重かったり頭痛がする →	○	○	○	○
22.首筋や肩がこる →	○	○	○	○
23.腰が痛い →	○	○	○	○
24.目が疲れる →	○	○	○	○
25.動悸や息切れがする →	○	○	○	○
26.胃腸の具合が悪い →	○	○	○	○
27.食欲がない →	○	○	○	○
28.便秘や下痢をする →	○	○	○	○
29.よく眠れない →	○	○	○	○

次へ

COPYRIGHT © iBRIDGE Corporation. All Rights Reserved.

6

あなたの仕事についてうかがいます．最もあてはまるものを選んでください． ***回答必須**

		1．そう思わない	2．ややそう思わない	3．どちらともいえない	4．ややそう思う	5．そう思う
自分のペースで仕事ができる	→	○	○	○	○	○
自分で仕事の順番・やり方を決めることができる	→	○	○	○	○	○
他の人々とやり取りをしながら進める必要がある	→	○	○	○	○	○

次へ

COPYRIGHT © iBRIDGE Corporation. All Rights Reserved.

7

あなたの仕事についてうかがいます．最もあてはまるものを選んでください． ***回答必須**

		1．そうだ	2．まあそうだ	3．ややちがう	4．ちがう
非常にたくさんの仕事をしなければならない	→	○	○	○	○

	1．そうだ	2．まあそうだ	3．ややちがう	4．ちがう
時間内に仕事を処理しきれない →	○	○	○	○
一生懸命働かなければならない →	○	○	○	○
かなり注意を集中する必要がある →	○	○	○	○
高度の知識や技術が必要なむずかしい仕事だ →	○	○	○	○
勤務時間中はいつも仕事のことを考えていなければならない →	○	○	○	○

次へ

COPYRIGHT © iBRIDGE Corporation. All Rights Reserved.

8

在宅勤務で使用しているご自宅の環境についてうかがいます．最もあてはまるものを選んでください．
在宅勤務時に使用している仕事場所の明るさはどの程度だと思いますか．　（ひとつだけ）*回答必須

- ○ 1.暗い
- ○ 2.やや暗い
- ○ 3.どちらともいえない
- ○ 4.やや明るい
- ○ 5.十分明るい

次へ

COPYRIGHT © iBRIDGE Corporation. All Rights Reserved.

9

在宅勤務時の周囲の環境音はどの程度だと感じますか.　（ひとつだけ）*回答必須

- 1.大きい
- 2.やや大きい
- 3.どちらともいえない
- 4.やや小さい
- 5.小さい

次へ

COPYRIGHT © iBRIDGE Corporation. All Rights Reserved.

10

在宅勤務時に使用している仕事場所の部屋の広さはどのように感じますか.　（ひとつだけ）*回答必須

- 1.狭い
- 2.やや狭い
- 3.どちらともいえない
- 4.やや広い
- 5.十分広い

次へ

COPYRIGHT © iBRIDGE Corporation. All Rights Reserved.

11

在宅勤務をするにあたってのご自身専用の机はありますか. （ひとつだけ）*回答必須

- ある
- ない（共用の机を使っている）
- 机は使用しない

次へ

COPYRIGHT © iBRIDGE Corporation. All Rights Reserved.

分岐条件

- **Q11**で**いずれかを選択した**選択肢： **[1.ある、2.ない（共用の机を使っている）]**

12

在宅勤務時に使用している机の広さはご自身の業務を行う上で十分な広さはありますか（ひとつだけ）*回答必須

- 1.狭い
- 2.やや狭い
- 3.どちらともいえない
- 4.やや広い
- 5.十分広い

次へ

COPYRIGHT © iBRIDGE Corporation. All Rights Reserved.

13

在宅勤務をするにあたってのご自身専用のイスはありますか．（ひとつだけ）*回答必須

- ある
- ない（共用のイスを使っている）
- イスは使用しない

次へ

COPYRIGHT © iBRIDGE Corporation. All Rights Reserved.

分岐条件

- **Q13**で**いずれかを選択した**選択肢：**［1.ある、2.ない（共用のイスを使っている）］**

14

在宅勤務時に使用しているイスに肘掛けはありますか．（ひとつだけ）*回答必須

- ある
- ない

次へ

COPYRIGHT © iBRIDGE Corporation. All Rights Reserved.

分岐条件

- **Q13でいずれかを選択した**選択肢： **[1.ある、2.ない（共用のイスを使っている）]**

15

在宅勤務時に使用しているイスに座面の高さなどを調整する機能はありますか. **（ひとつだけ）*回答必須**

- ある
- ない

次へ

COPYRIGHT © iBRIDGE Corporation. All Rights Reserved.

16

在宅勤務時のインターネット速度についてどのように感じますか. **（ひとつだけ）*回答必須**

- 1.非常に遅い
- 2.遅い
- 3.どちらともいえない
- 4.速い
- 5.非常に速い

次へ

COPYRIGHT © iBRIDGE Corporation. All Rights Reserved.

17

在宅勤務の際に使用しているPCの状況についてあてはまるものを選択してください．（ひとつだけ）*回答必須

- ノートPCを1台のみ使用している
- デスクトップPCを1台のみ使用している
- ノートPCを複数台併用している
- デスクトップPCを複数台併用している
- ノートPCとデスクトップPCを併用している

次へ

COPYRIGHT © iBRIDGE Corporation. All Rights Reserved.

18

在宅勤務の際に使用しているPCのスペックについてどのように感じますか．（ひとつだけ）*回答必須

- 1.十分なスペックがない
- 2.スペックがやや足りない
- 3.どちらともいえない
- 4.スペックは十分足りている
- 5.スペックは非常に高い

次へ

COPYRIGHT © iBRIDGE Corporation. All Rights Reserved.

19

在宅勤務の際に外付けのPCモニタを利用していますか.　（ひとつだけ）*回答必須

- している
- していない

次へ

COPYRIGHT © iBRIDGE Corporation. All Rights Reserved.

分岐条件

- **Q19**で**いずれかを選択した**選択肢： **[1.している]**

20

在宅勤務時に使用しているPCモニタに高さ調整の機能はありますか.　（ひとつだけ）*回答必須

- ある
- ない

次へ

COPYRIGHT © iBRIDGE Corporation. All Rights Reserved.

21

在宅勤務時に使用しているノートPCもしくは外付けPCモニタの画面サイズを教えてください．複数ある場合は，一番大きい画面のサイズをご回答ください．（ひとつだけ）*回答必須

- 13インチ以下程度
- 14～17インチ程度
- 18～22インチ程度
- 23～27インチ程度
- 28インチ以上

次へ

COPYRIGHT © iBRIDGE Corporation. All Rights Reserved.

22

在宅勤務時に以下の機器を利用していますか*回答必須

		している	していない
外付けスピーカー	→	○	○
外付けマイク	→	○	○
ヘッドセット	→	○	○

次へ

COPYRIGHT © iBRIDGE Corporation. All Rights Reserved.

23

在宅勤務時に使用している場所についてうかがいます．あてはまるものを選択してください． ***回答必須**

		ある	ない
在宅勤務用の専用スペースはありますか	→	○	○
在宅勤務時に使用している場所は，家事や普段の生活で使う部屋内にありますか	→	○	○
在宅勤務時に仕事用のスペース以外に食事や休憩ができるスペースがありますか	→	○	○

次へ

COPYRIGHT © iBRIDGE Corporation. All Rights Reserved.

排他
”特にいない” を選択した場合は他の項目、選択肢を答えることができません

24

以下に当てはまる同居者はいますか． **（いくつでも）*回答必須**

- ☐ パートナー
- ☐ 未就学児の子女

- [ ] 小学生の子女
- [ ] 中学生以上の学生の子女
- [ ] 学業を終えた子女
- [ ] 自分自身またはパートナーの親
- [ ] 特にいない

次へ

COPYRIGHT © iBRIDGE Corporation. All Rights Reserved.

分岐条件

- **Q24でいずれかを選択した選択肢： [1.パートナー～6.自分自身またはパートナーの親]**

25

在宅勤務時の同居者とのかかわりについてうかがいます．あてはまるものを選択してください． ***回答必須**

	1．ほとんどない	2．めったにない	3．ときどきある	4．よくある	5．とてもよくある
在宅勤務時に同居者が同じ部屋で仕事や勉強をすることはありますか →	○	○	○	○	○
在宅勤務中に同居者の都合や介入によって仕事を中断する頻度はどの程度ありますか →	○	○	○	○	○

次へ

COPYRIGHT © iBRIDGE Corporation. All Rights Reserved.

分岐条件

- Q24でいずれかを選択した選択肢： [1.パートナー～6.自分自身またはパートナーの親]

26

在宅勤務日における自分自身の家事分担の割合はどの程度ありますか． （ひとつだけ）*回答必須

- 1.ほとんど分担していない（同居者が行っている）
- 2.自分自身も家事は行っているが同居者の方が多く行っている
- 3.同居者とほぼ同程度家事を行っている
- 4.自分自身の家事分担量が多い
- 5.ほとんどすべて自分自身で行っている

次へ

COPYRIGHT © iBRIDGE Corporation. All Rights Reserved.

27

在宅勤務時の生活リズムについてうかがいます．あてはまるものを選択してください． *回答必須

1．ほとんどとっていない	2．あまりとっていない	3．どちらともいえない	4．ある程度とっている	5．ほぼ確実にとっている

	1.ほとんどとっていない	2.あまりとっていない	3.どちらともいえない	4.ある程度とっている	5.ほぼ確実にとっている
在宅勤務時に定期的な休憩はとっていますか →	○	○	○	○	○
在宅勤務時に決まった時間に食事をとっていますか →	○	○	○	○	○

次へ

COPYRIGHT © iBRIDGE Corporation. All Rights Reserved.

28

在宅勤務時に家事などによって仕事を中断する頻度はどの程度ありますか.　（ひとつだけ）*回答必須

- ○ 1.ほとんどない
- ○ 2.めったにない
- ○ 3.ときどきある
- ○ 4.よくある
- ○ 5.とてもよくある

次へ

COPYRIGHT © iBRIDGE Corporation. All Rights Reserved.

29

在宅勤務日の平均的な一日において家事に費やす時間はどの程度ありますか.　（ひとつだけ）*回答必須

- 1時間未満
- 1時間以上，2時間未満
- 2時間以上，3時間未満
- 3時間以上，4時間未満
- 4時間以上

次へ

COPYRIGHT © iBRIDGE Corporation. All Rights Reserved.

30

在宅勤務日の勤務時間（始業・終業の時間）は一定ですか.　（ひとつだけ）*回答必須

- 1.そう思わない
- 2.ややそう思わない
- 3.どちらともいえない
- 4.ややそう思う
- 5.そう思う

次へ

COPYRIGHT © iBRIDGE Corporation. All Rights Reserved.

31

勤務時間外のことについてうかがいます．あてはまるものを選択してください． *回答必須

	1．ほとんどない	2．めったにない	3．ときどきある	4．よくある	5．とてもよくある
在宅勤務日に勤務時間外に仕事のことを考えている時間はどの程度ありますか →	○	○	○	○	○
在宅勤務日に所定の勤務時間外に仕事をしてしまう頻度はどの程度ありますか →	○	○	○	○	○
休日に仕事のためにPCを見てしまう頻度はどの程度ありますか →	○	○	○	○	○

次へ

COPYRIGHT © iBRIDGE Corporation. All Rights Reserved.

32

在宅勤務の実施状況についてうかがいます．
ここ半年における平均的な1週間において自宅で勤務した日数はどのくらいありましたか． （ひとつだけ）
***回答必須**

- ○ 2日以下
- ○ 3日

- 4日
- 5日
- 6日以上

次へ

COPYRIGHT © iBRIDGE Corporation. All Rights Reserved.

33

ここ1か月の在宅勤務中におけるオンライン会議の1週間当たりの平均的な回数はどれくらいですか. （ひとつだけ）*回答必須

- 0~1回程度
- 2~4回程度
- 5~9回程度
- 10~14回程度
- 15回以上

次へ

COPYRIGHT © iBRIDGE Corporation. All Rights Reserved.

34

ここ1か月の在宅勤務中におけるオンライン会議の1日あたりの平均的な時間はどれくらいですか. （ひとつだけ）*回答必須

◯ 30分未満
◯ 30分以上，2時間未満
◯ 2時間以上，4時間未満
◯ 4時間以上，6時間未満
◯ 6時間以上

次へ

COPYRIGHT © iBRIDGE Corporation. All Rights Reserved.

35

ここ1か月の在宅勤務における平均的な1日の勤務時間はどれくらいですか. **（ひとつだけ）*回答必須**

◯ 4時間未満
◯ 4時間以上，6時間未満
◯ 6時間以上，8時間未満
◯ 8時間以上，10時間未満
◯ 10時間以上

次へ

COPYRIGHT © iBRIDGE Corporation. All Rights Reserved.

36

ここ1か月の在宅勤務における勤務時間中の平均的な1日の休憩時間はどれくらいですか. **（ひとつだけ）*回答必須**

- 30分未満
- 30分以上，1時間未満
- 1時間以上，1時間30分未満
- 1時間30分以上，2時間未満
- 2時間以上

次へ

COPYRIGHT © iBRIDGE Corporation. All Rights Reserved.

在宅勤務の状況および在宅勤務時の生活環境についてのアンケート

ご協力ありがとうございました。

アンケートは以上で終了です。
今回のアンケートの回答をもとに、今後のサービス向上の参考にさせて頂きます。

アンケート完了

※閉じない場合はブラウザから閉じてください。

COPYRIGHT © iBRIDGE Corporation. All Rights Reserved.
