# Supplementary material for: The relationship between the living environment and remote working: an analysis using the SHEL model
Source: PeerJ. 2024 May 7;12:e17301. doi: 10.7717/peerj.17301 (PMC11086296; doi:10.7717/peerj.17301)
Supplement: Supplemental Information 4 [file peerj-12-17301-s004.docx]

Age

Gender (1=Male)

Job (0=Managerial position)

Overall satisfaction

Satisfaction with the physical environment

Perceived productivity

Work engagement (Utrecht Work Engagement Scale (simplified version))

Stress reactions (Brief Job Stress Questionnaire)

Job autonomy (total score of the following three items)

- - 1. I can work at my own pace
    2. I can decide the order and method of work by myself
    3. I need to communicate with other people (inversion)

Qualitative workload (Brief Job Stress Questionnaire)

Quantitative workload (Brief Job Stress Questionnaire)

Brightness of workroom

Ambient noise

Size of workroom

Dedicated desk

Size of desk

Dedicated chair

Chair: armrests

Chair: adjustment

Internet speed

PC performance

External monitor

Size of PC monitor

External speaker

External microphone

Headset

Dedicated workspace

Workspace is within the space they use for housework and daily life

Space for eating and resting

Living with partner

Living with preschooler

Living with elementary school students

Living with junior high school students or older

Living with children who have completed their studies

Living with parents

Frequency of being in the same room with housemates

Frequency of intervention by housemates

Degree of housework sharing with housemates

Regularly breaks

Regularly meals

Frequency of interrupted work for household tasks

Time spent on household tasks

Regularly worktime

Frequency of thinking about work after work hours

Frequency of overtime working

Frequency of looking at a PC for work on days off

Number of telecommuting days per week

Number of online meetings per week

Online meeting hours per day

Telecommuting hours per day

Breaks per day
